# Construct validation of a DCM for resting state fMRI

**DOI:** 10.1016/j.neuroimage.2014.11.027

**Published:** 2015-02-01

**Authors:** Adeel Razi, Joshua Kahan, Geraint Rees, Karl J. Friston

**Affiliations:** a The Wellcome Trust Centre for Neuroimaging, University College London, 12 Queen Square, London WC1N 3BG, UK; b Department of Electronic Engineering, NED University of Engineering and Technology, Karachi, Pakistan; c Sobell Department of Motor Neuroscience & Movement Disorders, Institute of Neurology, University College London, Queen Square, London, WC1N 3BG, UK; d Institute of Cognitive Neuroscience, University College London, 17 Queen Square, London WC1N 3AR, UK

**Keywords:** Dynamic causal modelling, Effective connectivity, Functional connectivity, Resting state, fMRI, Graph, Bayesian, Network discovery

## Abstract

Recently, there has been a lot of interest in characterising the connectivity of resting state brain networks. Most of the literature uses functional connectivity to examine these intrinsic brain networks. Functional connectivity has well documented limitations because of its inherent inability to identify causal interactions. Dynamic causal modelling (DCM) is a framework that allows for the identification of the causal (directed) connections among neuronal systems — known as effective connectivity. This technical note addresses the validity of a recently proposed DCM for resting state fMRI – as measured in terms of their complex cross spectral density – referred to as spectral DCM. Spectral DCM differs from (the alternative) stochastic DCM by parameterising neuronal fluctuations using scale free (i.e., power law) forms, rendering the stochastic model of neuronal activity deterministic. Spectral DCM not only furnishes an efficient estimation of model parameters but also enables the detection of group differences in effective connectivity, the form and amplitude of the neuronal fluctuations or both. We compare and contrast spectral and stochastic DCM models with endogenous fluctuations or state noise on hidden states. We used simulated data to first establish the face validity of both schemes and show that they can recover the model (and its parameters) that generated the data. We then used Monte Carlo simulations to assess the accuracy of both schemes in terms of their root mean square error. We also simulated group differences and compared the ability of spectral and stochastic DCMs to identify these differences. We show that spectral DCM was not only more accurate but also more sensitive to group differences. Finally, we performed a comparative evaluation using real resting state fMRI data (from an open access resource) to study the functional integration within *default mode network* using spectral and stochastic DCMs.

## Highlights

• This paper provides construct validation of spectral DCM against stochastic DCM.
• Spectral DCM is shown to be more accurate than stochastic DCM in terms of root mean square error.
• Spectral DCM is shown to be more sensitive at identifying group differences.

## Introduction

In recent years there has been a marked increase in research that combines resting state fMRI with large-scale ‘network analyses’ (Nakagawa et al., 2013; Smith et al., 2013; Wang et al., 2010). The majority of studies report functional connectivity, reflecting statistical dependencies (e.g. temporal correlations) between spatially remote neurophysiological regions. These correlations are inherently undirected, and preclude inference about (directed) causal interactions among neuronal systems. In contrast, effective connectivity summarises the causal influence one neural system exerts over another using a model of neural interactions that best explains the observed signals or their functional connectivity: in brief, effective connectivity causes functional connectivity. Effective connectivity is, necessarily, directed. However, it should be noted that functional connectivity can also be directed; e.g., partial correlation and parametric (resp. nonparametric) Granger causality based on Yule–Walker (resp. Wilson–Burg) formulations. These characterisations provide measures of directed statistical dependencies because they consider how much past observations predict the current observation (Friston et al., 2014a).

We recently introduced a new dynamic causal model (DCM) for modelling intrinsic dynamics of a resting state network (Friston et al., 2014b). This DCM estimates the effective connectivity among coupled populations of neurons, which subtends the observed functional connectivity in the frequency domain. We refer to this as spectral DCM (spDCM). Spectral DCM uses a neuronally plausible power-law model of the coupled dynamics of neuronal populations to generate complex cross spectra among measured responses. In particular, this spectral DCM is distinct from stochastic DCM (sDCM) (Friston et al., 2011; Li et al., 2011) as it eschews the estimation of random fluctuations in (hidden) neural states; rendering spectral DCM essentially deterministic in nature.

In this paper, we establish the construct validity of spectral DCM by comparing and contrasting it with stochastic DCM. This paper comprises of four sections. We begin by rehearsing the theoretical background of both spectral and stochastic dynamic causal models. These models are similar to conventional deterministic DCM for fMRI (Friston et al., 2003) but include these statistics of endogenous activity that reproduce functional connectivity (correlations) — of the sort observed in resting state fMRI. DCMs for resting state data are also slightly simpler: given that most resting state designs compare groups of subjects (e.g. patient cohorts vs. controls); spDCMs do not usually require the bilinear term (accounting for condition-specific effects on effective connection strengths). In the second section, we address construct validation of spectral DCM against stochastic DCM, using simulated time series. We compare the accuracy of model inversion, demonstrating that ‘true’ effective connectivity can be recovered using both schemes with an acceptable root mean square error. In the third section, we repeat the simulations but with simulated group differences, to see if the schemes can successfully recover true differences in effective connectivity. In the fourth section, we conclude with an empirical (comparative) evaluation using data available from an open source database, with an illustrative focus on functional integration within the default mode network.

## Dynamic casual modelling of resting brain networks

There has been a large surge of research recently examining spontaneous fluctuations in Blood Oxygen Level Dependent (BOLD) fMRI signals measured using fMRI. These fluctuations can be attributed to the spontaneous neural activity, which is usually ignored in the deterministic DCM models of responses to (designed) experimental inputs.

Classically, deterministic DCMs are cast as multiple input multiple output (MIMO) systems, where the experimentally designed inputs enter the brain to produce an observed BOLD response. In the absence of external inputs – as is the case in the resting state fMRI – one would imagine that neuronal networks are perturbed by activity that is internal to the system or by intrinsic fluctuations seen in any biological system for e.g. see Faisal et al. (2008). These perturbations are the endogenous neural fluctuations, which are responsible for driving the coupled dynamics of the hidden neuronal states. The inclusion of the neural fluctuations means that the model is now based on random differential equations having both the drift and diffusion components. The drift component of this random differential equation entails the Jacobian; i.e., the effective connectivity matrix, whilst the diffusion component models the endogenous fluctuations. We have introduced several schemes that can invert these sorts of models (Friston et al., 2010, 2014b; Li et al., 2011) that differ according to how the endogenous fluctuations are treated.

Model inversion using sDCM estimates both the effective connectivity and the neuronal fluctuations from the BOLD signal, which entails a difficult triple estimation problem and uses advance methods like *generalised filtering* (Friston et al., 2010) for parameter estimation. In contrast, spDCM parameterises the form of the endogenous fluctuations; specifically, by characterising them in terms of their cross spectral densities. This eliminates the need to estimate the neuronal fluctuations and significantly simplifying model inversion. In what follows, we briefly review these models in turn. It is useful to remember that DCM for fMRI consists of two parts; (1) the neuronal state model, describing how the dynamics of coupled neuronal populations interact, and (2) the haemodynamic model, which transforms hidden neural states of each population or region into predicted BOLD responses — using a previously established biophysical model (Buxton et al., 1998; Friston et al., 2003; Stephan et al., 2007). In this paper, we are only concerned with the first (neuronal) part, without changing the second (haemodynamic) part: see Stephan et al. (2007).

### The generative model

In modelling resting state activity, it is necessary to augment the ordinary differential equations used in standard DCM, with a stochastic term to model endogenous neuronal fluctuations. This renders the equations of the motion stochastic. Stochastic equations are most frequently used to model the behaviour of (open) systems operating near or far from equilibrium that are perturbed by fluctuations in the (thermal) environment. The stochastic generative model for the resting state fMRI time series, like any other DCM, comprises of two equations: the Langevin form of evolution equation (motion) is written as:

(1) $$\dot{x}\left(t\right)=f\left(x\left(t\right),u\left(t\right),\theta \right)+\;v\left(t\right)\text{,}$$

and the observation equation, which is a static nonlinear mapping from the hidden physiological states^1^ in (1) to the observed BOLD activity and is written as:

(2) $$y\left(t\right)=h\left(x\left(t\right),\varphi \right)+\;e\left(t\right)\text{,}$$

where *ẋ*(t) is the rate in change of the neuronal states *x*(*t*), *θ* are unknown parameters (i.e. the effective connectivity) and *v*(*t*) (resp. *e*(*t*)) is the stochastic process – called the state noise (resp. the measurement or observation noise) – modelling the random neuronal fluctuations that drive the resting state activity. In the observation equations, *φ* are the unknown parameters of the (haemodynamic) observation function and *u*(*t*) represents any exogenous (or experimental) inputs that drive the hidden states — that are usually absent in resting state designs. Here, we will assume a generalised framework in which *v*(*t*) and *e*(*t*) are analytic (i.e., non-Markovian). This simply means that *generalised* motion of the state noise $\tilde{v}\left(t\right)=\left[v\left(t\right),\dot{v}\left(t\right),\ddot{v}\left(t\right)\ldots \right]$ is well defined in terms of its covariance. Similarly, the observation noise *ẽ*(*t*) = [*e*(*t*), *ė*(*t*), *ë*(*t*) …] has well defined covariance (for a more detailed discussion see Friston, 2008). Consequently, the diffusion part of the generative model above can be conveniently parameterised in terms of its precision (inverse covariance). This allows us to cast (1) as random differential equation instead of stochastic differential equation hence eschewing Ito calculus (Friston et al., 2010; Li et al., 2011).

Statistical assumptions about the higher order motion of generalised state noise implicitly specify its degree of smoothness. Interested readers will find a theoretical motivation for using analytic state noise in the context of studying brain dynamics in (Friston et al., 2010). Note that standard stochastic differential equations (and related Ito calculus) rely upon the fluctuations being a mixture of Wiener processes, which are non-differentiable functions of time (Kloeden and Platen, 1999). This corresponds to a special case of generalised state noise whose high order motion has infinitely high prior variance (unbounded roughness). Within the above class of generalised state-space models, standard Markovian state-space models are thus rather special (and biologically implausible) cases of generalised models.

Under linearity assumptions, Eqs. (1) and (2) can be written compactly in generalised coordinates of motion:

(3) $$D\tilde{x}\left(t\right)=\tilde{f}\left(\tilde{x},\tilde{u},\theta \right)+\;\tilde{v}\left(t\right)\text{,}$$

(4) $$\tilde{y}\left(t\right)=\tilde{h}\left(\tilde{x},\varphi \right)+\;\tilde{e}\left(t\right)\text{,}$$

where *D* is the block diagonal temporal derivative operator, such that the vectors of generalised coordinates of motion are shifted as we move from lower-orders of motion to higher-orders. This means that the first leading diagonal of *D* contains identity matrices. For a more detailed derivation and explanation please see Friston (2008). For resting state activity, Eq. (3) takes on a very simple linear form:

(5) $$D\tilde{x}\left(t\right)=A\tilde{x}\left(t\right)+C\tilde{u}\left(t\right)+\;\tilde{v}\left(t\right)\text{,}$$

where *A* is the Jacobian describing the behaviour – i.e. the effective connectivity – of the system near its stationary point (*f*(*x*_*o*_) = 0) in the absence of the fluctuations *ṽ*(*t*). Eq. (5) is an instance of a linear dynamical system and has quasi-deterministic behaviour (Daunizeau et al., 2012; Tropper, 1977). Put simply, the linear dynamical system described by Eq. (5) is insensitive to the initial conditions; hence, it can only exhibit a limited repertoire of behaviour: linear systems can contain closed orbits, but they will not be *isolated*, hence no limit cycles – either stable or unstable – can exist, which precludes chaotic behaviour. Technically speaking, if *λ* represents the eigenvalues of the Jacobian $\partial_{\tilde{x}}f=A$, that is *λ* = *ν*^†^*Aν*, where † denotes the generalised inverse, then the Lyapunov exponents *ℜ*(*λ*) of this linear dynamical system will always be negative. In general, the Jacobian is not symmetrical (causal effects are asymmetric); hence the modes and eigenvalues take complex values. For the detailed treatment of the special case of symmetrical connectivity – in which the eigenmodes of functional and effective connectivity become the same – see Friston et al. (2014c). The real part of the eigenvalues plays the role of Lyapunov exponents, whereas the imaginary part represents the oscillatory portion of the response, which will decay (or grow) with the real part. In terms of dynamical behaviour, irrespective of the amount of the stochastic noise, the average trajectory of the stochastic system would follow the trajectory of its deterministic variant — in the absence of any stochastic forcing term.

### Dynamic instabilities and intrinsic brain networks

Although the resting state model of effective connectivity is simple – and admits only a limited repertoire of dynamical behaviour – the inclusion of endogenous fluctuations provides a plausible model of intrinsic brain networks: intrinsic dynamics are thought to be generated by the dynamic instabilities that occur near *bifurcations*; i.e., dynamics that accompany a loss of stability when certain control parameter(s) reach a critical value (Deco and Jirsa, 2012; Freyer et al., 2011; Robinson et al., 2002). The eigenmodes of effective connectivity that define the stability of the resting state give rise to scale-free fluctuations that emerge from the superposition of the few modes that decay slowly. These slowly fluctuating (unstable) modes have Lyapunov exponents that are close to zero. This occurs when systems approach *transcritical* bifurcations (or stochastic Hopf bifurcations) when the eigenvalues are complex (Aburn et al., 2012; Robinson, 2003) and show critical slowing (Kelso, 2010). This simply means that the ensuing networks are defined by trajectories that have a fixed point that always exists but which is also close to instability. This means that the neural fluctuations persist over longer time scales to generate the patterns responsible for the emergence of intrinsic brain networks. The (negative) inverse of the Lyapunov exponent corresponds to the characteristic time constant of each mode, where each mode corresponds to an intrinsic brain network.

### Free energy and Bayesian model inversion

Having considered DCMs of resting state activity, we turn to model inversion. Bayesian model inversion means that we infer the parameters of the model in Eqs. (3) and (4), from the observed signal *y*(*t*). Since this inversion is computationally exorbitant, requiring high-dimensional integrals (either by brute force numerical methods or sampling schemes), we resort to schemes based on approximate *variational Bayesian* inference. Variational Bayes approximates the conditional posterior density *p*(*ψ*|*y*, *m*) – of model parameters *ψ* with data *y* for a given model *m* – by a variational or proposal density *q*(*ψ*). Importantly, this approximation is optimised by maximising model log-evidence, which can be expressed mathematically as

(6) $$\ln p\left(y|m\right)=F\left(y,q\right)+D_{KL}\left[q\left(\psi \right)||p\left(\psi |y,m\right)\right]\text{,}$$

where *F*(*y*, *q*) is the *free energy* and *D_KL_* is the Kullback–Leibler divergence. In turn, free energy can be expressed as

(7) $$\begin{array}{c} F\left(y,q\right)=E_{q}\left[\ln p\left(\psi ,y|m\right)]\;–\;E_{q}[\ln q\left(\psi \right)\right],\\ =E_{q}\left[\ln p\left(y|\psi ,m\right)+\ln p\left(\psi |m\right)\right]–\;E_{q}\left[\ln q\left(\psi \right)\right]\text{,} \end{array}$$

where $E_{q}\left[\text{.}\right]$ is the expectation operator with respect to *q*(*ψ*). There are few important observations to make here: Firstly, from Eq. (6) free energy *F*(*y*, *q*) ≤ ln *p*(*y*|*m*) is always a lower bound on the model log evidence because the divergence term is always positive and only becomes zero when *q*(*ψ*) = *p*(*ψ*|*y*, *m*). Secondly, maximising *F*(*y*, *q*) automatically minimizes the divergence term hence giving *q*(*ψ*) ≈ *p*(*ψ*|*y*, *m*). In other words, by maximising negative free energy with respect to the variational density we get two things; first, the free energy becomes a proxy for log model evidence (necessary for model comparison) and second, the variational density becomes posterior density over parameters (necessary for model identification).

Under the Laplace assumption, the proposal density assumes a Gaussian form $q\left(\psi \right)=N\left(\mu ,\Sigma \right)$, where the variational parameters, *μ* and Σ, corresponds to the conditional mean and covariance respectively. The free energy from Eq. (7) – under Laplace approximation – can now be written as

(8) $$F\left(y,q\right)=\ln p\left(y|\mu ,m\right)+\ln p\left(\mu |m\right)+\;\frac{1}{2}\left[\ln|\Sigma |\;+n\;\ln2\pi e\right]\text{,}$$

where *n* is the number of the parameters in the set *ψ*.

Another assumption that simplifies the maximisation of free energy *F*(*y*, *q*) is the mean field assumption used in statistical physics. This means that one can factorise the proposal density over a set of parameters such that

(9) $$q\left(\psi \right)=\prod_{i}q\left(\psi^{i}\right)\text{.}$$

In other words, *F*(*y*, *q*) is maximised with respect to *q*(*ψ^i^*) when there is no variation in free energy with respect to *q*(*ψ^i^*) (Friston et al., 2007). Mathematically this can be written as:

(10) $$\begin{array}{l} \Delta F\left(y,q\left(\psi^{i}\right)\right)=0\Leftrightarrow \frac{\partial F\left(y,q\left(\psi^{i}\right)\right)}{\partial q\left(\psi^{i}\right)}=0\Rightarrow \\ q\left(\psi^{i}\right)=\frac{1}{Z_{i}}\exp\left(I\left(q\left(\psi^{i}\right)\right)\right)\text{,} \end{array}$$

where *Z_i_* is a normalization constant and *I*(*q*(*ψ*^*i*^)) is the variational energy. In summary, model inversion reduces to maximising the free energy in Eq. (8) with respect to the mean (and covariance) of the proposal density over each set of parameters. DCM generally uses a gradient ascent scheme, known as the Variational Laplace (VL).

### Inversion of stochastic models in time domain

Inverting the stochastic DCM of the form given by Eq. (1) in the time domain, which includes state noise, is rather complicated because such models require estimation of not only the model parameters (and any hyperparameters that parameterise the random fluctuations), but also the hidden states, which become random (probabilistic) variables. Hence the unknown quantities to be estimated under a stochastic DCM are $\psi =\left\{\tilde{x}\left(t\right),\varphi ,\;\theta ,\sigma \right\}$, where *σ* refers to any hyperparameters (precisions or inverse covariances) defining the neuronal fluctuations. In terms of temporal characteristics, the hidden states are time-variant, whereas the model parameters (and hyperparameters) are time-invariant. There are various variational schemes in literature that can invert such models, for example, dynamic expectation maximisation (DEM) (Friston et al., 2008) and generalised filtering (GF) (Friston et al., 2010). There is a subtle but important distinction between DEM and GF. DEM calls on the mean field approximation described above i.e., it assumes $q\left(\psi \right)=q\left(\tilde{x}\left(t\right)\right)q\left(\varphi \right)q\left(\theta \right)q\left(\sigma \right)$, whereas generalised filtering, as the name suggest, is more general in a sense that it does not make this assumption. Both schemes, however, assume a fixed form Gaussian distribution for the approximate conditional posterior densities (the Laplace approximation). Generalised filtering considers all unknown quantities to be conditionally dependant variables i.e., $q\left(\psi \right)=q\left(\tilde{x},\varphi ,\theta ,\sigma \right)$, and furnishes time-dependent conditional densities for all the unknown quantities. The time-*invariant* parameters and hyperparameters are cast as time-variant with the prior constraint that their temporal variation is very small. This translates into a smooth gradient ascent on the free energy landscape. In brief, this online scheme assimilates log-evidence at each time point, in the form of the free energy bound and provides time-dependant conditional densities for all unknown quantities. This is in contrast to schemes, like DEM (or deterministic model inversion using VL) with mean field approximations that assimilates all the data before computing the free energy. The marginal conditional densities for the time-averaged parameters (and hyperparameters) from generalised filtering (GF) are calculated by using Bayesian parametric averaging (BPA). This allows one to compare GF with other schemes like the deterministic model inversion employed in spDCM.

### Inversion of stochastic models in spectral domain

Although the stochastic models in Eq. (1) and their inversion in time domain provide a useful means to estimate effective connectivity they also require us to estimate hidden states. This poses a difficult inverse problem that is computationally demanding; especially when the number of hidden states becomes large. To finesse this problem, we recently introduced a DCM based upon a deterministic model that generates predicted cross spectra (Friston et al., 2014b). This scheme furnishes a constrained inversion of the stochastic model by parameterising the neuronal fluctuations. This parameterisation also provides an opportunity to compare parameters encoding the neuronal fluctuations among groups. The parameterisation of endogenous fluctuations means that the states are no longer probabilistic; hence the inversion scheme is significantly simpler, requiring estimation of only the parameters (and hyperparameters) of the model. The ensuing model inversion in the spectral domain is similar in spirit to previous approaches described in (Freyer et al., 2012; Robinson, 2003; Robinson et al., 2004). Put simply, whilst DEM or GF estimates time-dependent fluctuations in neuronal states producing observed fMRI data, spDCM simply estimates the time-invariant parameters of their cross spectra. Effectively, this is achieved by replacing the original time series with their second-order statistics (i.e., cross spectra), under stationarity assumptions. This means that, instead of estimating time varying hidden states, we are estimating their covariance, which does not change with time. This means that we need to estimate the covariance of the random fluctuations; where a scale free (power law) form for the state noise (resp. observation noise) that can be motivated from previous work on neuronal activity (Beggs and Plenz, 2003; Shin and Kim, 2006; Stam and de Bruin, 2004):

(11) $$\begin{array}{l} g_{v}\left(\omega ,\theta \right)=\alpha_{v}\omega^{-\beta_{v}}\\ g_{e}\left(\omega ,\theta \right)=\alpha_{e}\omega^{-\beta_{v}}\text{.} \end{array}$$

Here, {*α*, *β*} ⊂ *θ* are the parameters controlling the amplitudes and exponents of the spectral density of the neural fluctuations. This models neuronal noise with generic *1*/*f^γ^* spectra, which characterises fluctuations in systems that are at nonequilibrium steady-state. A linear scaling regime of the spectral density in double logarithmic coordinates –implicit in (11) – is not by itself indicative of a scale free, critical process unless *γ* is less than 1.5 — and the regime scales over several orders of magnitude. We note that for the human EEG, this is generally not the case – above 10 Hz, *γ* = 2.5 and above 70 Hz *γ* is usually greater than 3.5 – which is consistent with a Poisson process (see Bedard et al., 2006; Miller et al., 2009). At low frequencies (less than 1.5 Hz) the slope is shallower and it is likely that the amplitude or power envelopes of faster frequencies are scale-free (Kitzbichler et al., 2009; Linkenkaer-Hansen et al., 2001) or heavy-tailed (Freyer et al., 2009).

Using the model parameters, *θ* ⊇ {*A*, *C*, *α*, *β*}, we can simply generate the expected cross spectra:

(12) $$\begin{array}{l} y\left(t\right)=\kappa \left(t\right)⊗v\left(t\right)+e\left(t\right),\\ \kappa \left(t\right)=\partial_{x}g\;\exp\left(t\;\partial_{x}f\right),\\ g_{y}\left(\omega ,\theta \right)={\left|K\left(\omega \right)\right|}^{2}g_{v}\left(\omega ,\theta \right)+g_{e}\left(\omega ,\theta \right)\text{,} \end{array}$$

where *K*(*ω*) is the Fourier transform of the system's (first order) Volterra kernels *κ*(*t*), which are a function of the Jacobian or effective connectivity. The unknown quantities *ψ* = {*φ*, *θ*, *σ*} of this deterministic model can now be estimated using standard Variational Laplace procedures (Friston et al., 2007). The resulting inversion provides the free energy bound on the log evidence ln *p*(*g*_*y*_(*ω*)|*m*) and approximate conditional densities *q*(*ψ*) ≈ *p*(*ψ*|*g*(*ω*), *m*). Here *g_γ_*(*ω*) represents the predicted cross spectra that can be estimated, for example, using autoregressive (AR) model. Specifically, we use a fourth-order autoregressive model to ensure smooth sample cross spectra of the sort predicted by the generative model. The frequencies usually considered for fMRI range from $\frac{1}{128}\;\mathrm{Hz}$ to the Nyquist frequency (half the sampling rate) in 32 evenly spaced frequency bins.

In summary, both sDCM and spDCM furnish estimates of the effective connectivity of endogenous brain networks from BOLD data acquired at rest, using different inversion schemes. We suppose that these resting state networks emerge from the dynamical instabilities and critical slowing near transcritical bifurcations. Hidden neuronal activity is modelled with random differential equations, which can be estimated using stochastic inversion schemes (like generalised filtering in sDCM), or by deterministic scheme modelling observed functional connectivity (specifically the cross spectral densities in the case of spDCM). In what follows, we use Monte Carlo simulations to assess the performance of these schemes.

## Comparative inversions and face validity

**Fig. 1:**
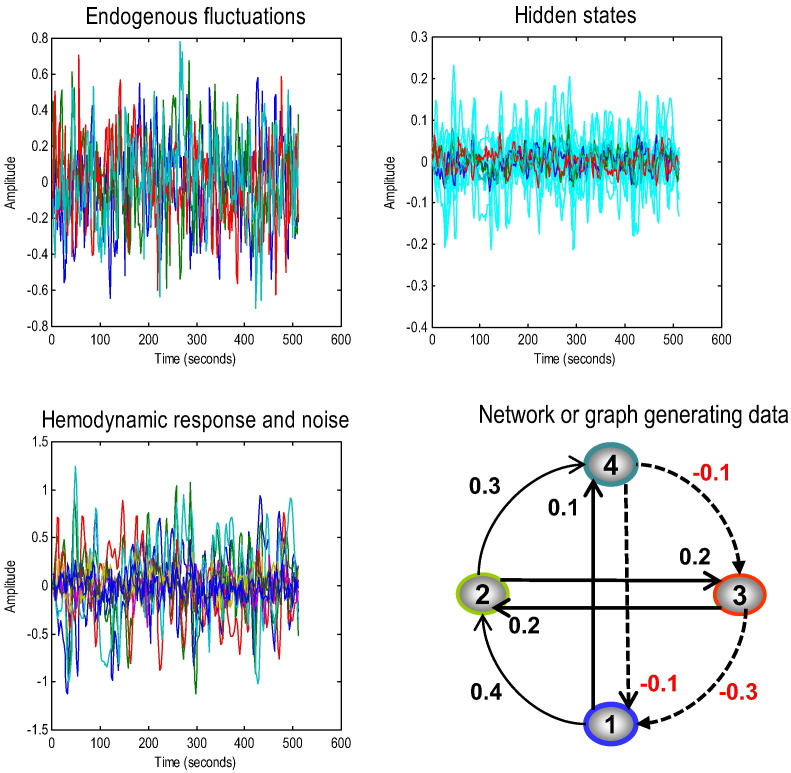
This figure summarises the results of simulating fMRI responses to endogenous fluctuations over 512 time points (scans) with a TR of 2 s — here we only show initial 256 time bins. The simulation was based upon a simple four-region hierarchical network or graph, shown on the lower right, with positive effective connectivity (black) in the forward or ascending direction (and lateral direction) and negative (red) in the backward or descending direction. The four regions were driven by endogenous fluctuations (upper right panel) generated from an AR(1) process with autoregressive coefficient of one half (and scaled to a standard deviation of one quarter). These fluctuations caused distributed perturbations in neuronal states and consequent changes in haemodynamic states (shown as cyan) in the upper right panel, which produce the final fMRI response in the lower left panel.

In this section, we address the face validity of the two schemes, comparing the models based on deterministic and stochastic modelling of neuronal fluctuations. Simulated time series were generated from a four node graph (producing data over 512 time bins with a repetition time of 2 s) with known effective connectivity (see Eq. (13) and Fig. 1). Smooth neuronal fluctuations (resp. observation noise) driving each node were independently generated based on an AR(1) process with an autoregressive coefficient of one half, scaled to a standard deviation of one fourth (resp. one eighth). The equations of motion in Eq. (1), together with haemodynamic observation Eq. (2) were used to generate synthetic slow-varying time series, reminiscent of BOLD data acquired at rest (Fig. 1). With these parameters, we produce a maximum fMRI signal change of around 2%. The upper panels in Fig. 1 show the variations in the amplitude of endogenous fluctuations that drive the changes in the hidden and haemodynamic states (cyan), which in turn produce the observed BOLD response. It is worth noting that the haemodynamic signal is much smoother than the neuronal variations – that reflect the low-pass filter-like effect of the haemodynamic transfer function – with a time constant of several seconds.

To assess the face validity of both schemes, we used sDCM and spDCM to estimate the underlying effective connectivity of the same data. For this, we used the usual priors for the hidden states, parameters (and hyperparameters) for stochastic DCM as described previously (Li et al., 2011), as well as the same priors for {*A*, *α*, *β*} in spectral DCM as described in (Friston et al., 2014b). The effective connectivity matrix used for generating the simulations had four nodes with bottom-up (i.e. forward) and lateral connections having positive coupling, and the top-down edges having negative coupling (lower right panel of Fig. 1). This architecture corresponds to the directed and cyclic connectivity matrix:

(13) $$A=\left[\begin{array}{cccc} -0.5 & 0 & -0.3 & -0.1\\ 0.4 & -0.5 & 0.2 & 0\\ 0 & 0.2 & -0.5 & -0.1\\ 0.1 & 0.3 & 0 & -0.5 \end{array}\right]\text{.}$$

Note that the equations of motion given by Eq. (5) — only yield critical neural fluctuations when the principal eigenvalues of the Jacobian (i.e., *A*) are close to zero (see Spasojevic et al., 1996). The effective connectivity matrix chosen here does not meet this criterion because of the strongly damped self-connections — which are usually fixed (a priori) at − 0.5 to preclude any run-away excitation.

**Fig. 2:**
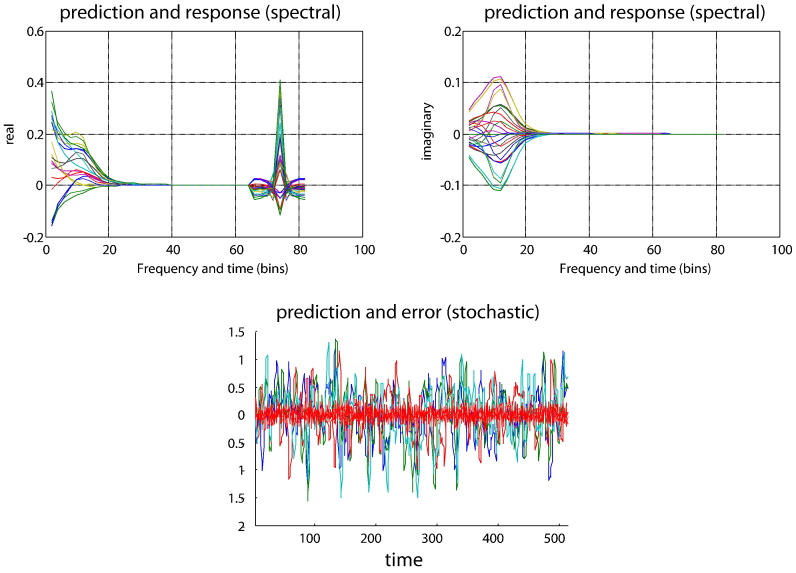
This figure reports the results of Bayesian model inversion using data shown in the previous figure. This inversion produced predictions (solid lines) of sample cross spectra (dashed lines) and cross covariance functions, shown in the upper two panels for spectral DCM. The real values are shown on the left and the imaginary values on the right. Imaginary values are produced only by extrinsic (between regions) connections. The first half of these responses and predictions correspond to the cross spectra between all pairs of regions, whilst the second half are the equivalent cross covariance functions — note that cross covariance only has real part. The lower panel shows the predicted response, in time, for the four regions and the associated error between the predictions and the observed responses.

The ensuing data we used to recover the known connection strengths in Eq. (13) using the original time series (for stochastic DCM) or their sample cross spectral density (for spectral DCM). The predicted and observed data features are presented in Fig. 2. The sampled (dashed lines) and predicted (solid lines) cross spectra of the data using spDCM were well matched. The upper left and right panels show the real and imaginary parts of the cross spectra respectively, whilst the second half of the upper left panel includes the cross covariance function (which is real in nature). The lower panel reports the predicted time series for each region (conditional expectations — solid lines) and errors (red dashed lines) from the sDCM inversion. As previously demonstrated (Li et al., 2011), the prediction error remained low, generating a predicted time series that very much resembled the observed data.

**Fig. 3:**
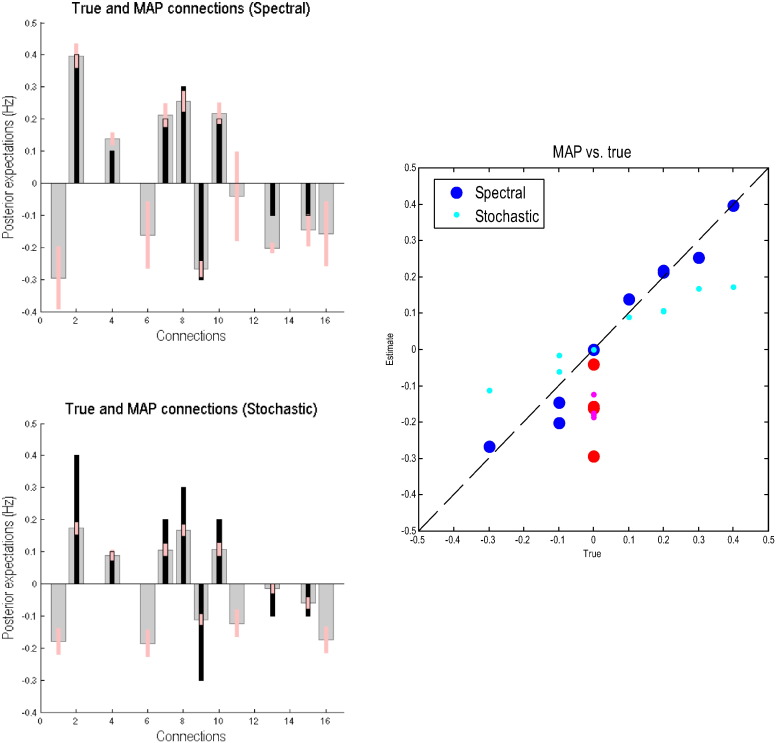
This figure shows the posterior estimates that result from the Bayesian inversion of the simulated time series. The posterior means (grey bars) and 90% confidence intervals (pink bars) are shown with the true values (black bars) in the left column. The upper and lower panel reports spectral and stochastic DCMs respectively. The grey bars depict the posterior expectations of connections, where intrinsic (within region) or self-connections are parameterised in terms of their log scaling (such that a value of zero corresponds to a scaling of one). The extrinsic (between regions) connections are measured in Hz in the usual way. It can be seen that, largely, the true values fall within the Bayesian confidence intervals for spectral DCM but not for stochastic DCM. The right panel shows the same results but plotting the estimated connection strengths against their true values. For spectral DCM (resp. stochastic DCM), the blue (resp. cyan) circles correspond to extrinsic connections and the red (resp. magenta) circles to intrinsic connectivity.

The posterior estimates of the effective connectivity are shown in Fig. 3. Posterior expectations are presented as grey bars with pink bars indicating the 90% Bayesian confidence intervals. We have also superimposed the true connectivity as black bars for comparison. The upper left panel shows the posterior expectations for the spDCM inversion (shown as spectral in title), whilst the lower left panel shows the results for the stochastic scheme using generalised filtering. Clearly, the spectral DCM's estimates are very accurate, with most of the extrinsic connection strengths falling within 90% confidence intervals. The intrinsic connections (i.e. the self-connections) are modelled as a (log) scale parameter and have a prior mean of zero. These connections are still estimated with good accuracy showing around a 20% underestimation of the self-connectivity. The stochastic scheme also performed well, with estimates tending towards the true values but not as accurately as the deterministic (spectral) scheme. This reiterates the point that stochastic DCM can find it difficult to recover effective connectivity from data generated from graphs with reciprocal connectivity. We have also presented these results in a scatter plot to illustrate the relative accuracies of the spectral and stochastic estimates (the posterior estimates of spDCM are closer to the true parameters than those generated by the model based on sDCM). It can also be seen that the stochastic model underestimates the parameters, a behaviour which has previously been reported (Li et al., 2011) and is generally characteristic of approximate Bayesian inference schemes that contend with conditional dependencies.

## Estimation accuracy and session length

In this section, we examine the accuracy of the estimates from the two DCMs as a function of the number of time bins sampled. It has previously been shown that including neural state noise yields more accurate estimates for sDCM compared to its deterministic version – which was extended to account for neural fluctuations by projecting the state noise to some temporal basis functions – especially so in the presence of nonlinearities (Daunizeau et al., 2012). The spectral DCM differs from earlier deterministic formulations; here we are parameterising the neuronal state noise in frequency domain meaning, in principle, we are still using the same stochastic model but now the model inversion uses second order data statistics — like their cross spectra.

When assessing the accuracy of the spectral and stochastic versions, we were primarily interested in establishing whether both versions can predict network parameters with an acceptable accuracy, whilst acknowledging their respective differences. The accuracy of sDCM has been assessed previously (Daunizeau et al., 2012; Li et al., 2011). Here the objective is to compare and contrast the accuracy of stochastic and spectral schemes. For this purpose we use the statistical measure of root mean squared error:

(14) $${RMS}_{\theta }=\;\sqrt{\frac{1}{n_{\theta }}\sum_{j=1}^{n_{\theta }}{\left(\theta_{j}-{\hat{\theta }}_{j}\right)}^{2}}\text{,}$$

where $\theta \;\mathrm{and}\;\hat{\theta }$ are the true and estimated parameter vectors respectively, and *nθ* is the length of these vectors. As argued in Daunizeau et al. (2012), the (root) mean squared error is an optimal measure of estimation accuracy — in terms of Bayesian decision theory.

**Fig. 4:**
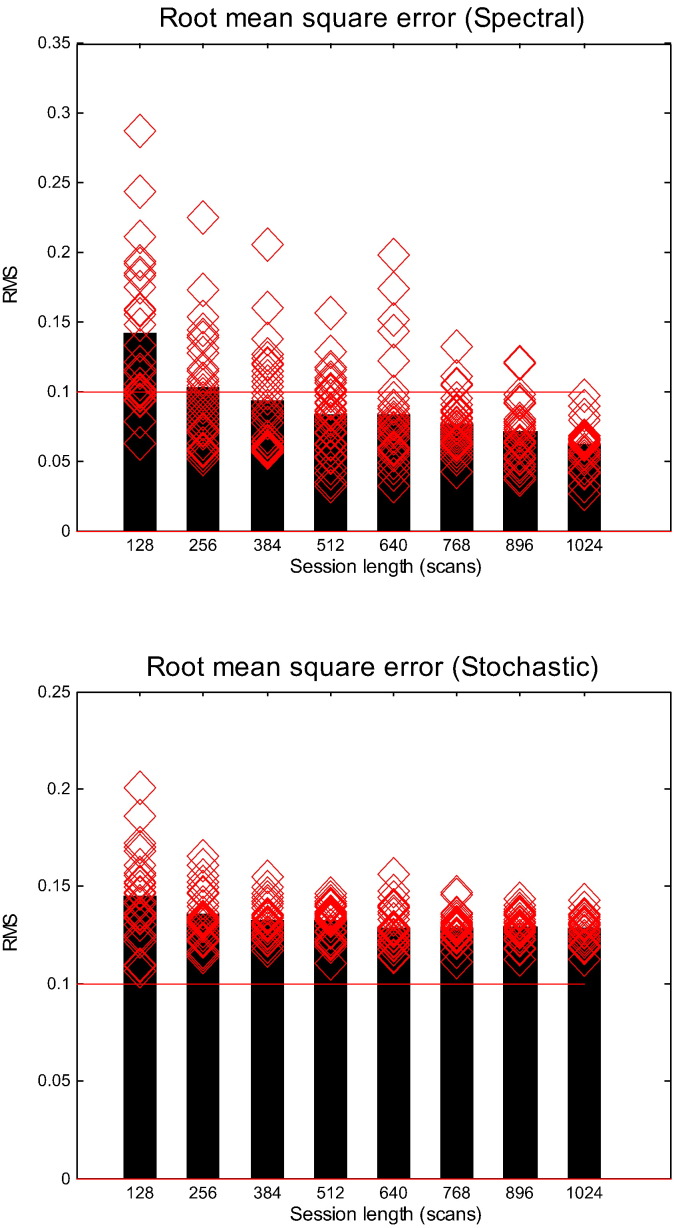
This figure reports the results of Monte Carlo simulations assessing the accuracy of posterior estimates in terms of root mean square error (RMS) from the true value. Both panels show the results of 32 simulations (red diamonds) for different run or session lengths. For the upper panel – that reports the results for spectral DCM – the average root mean square error (black bars) decreases with increasing run length to reach acceptable (less than 0.1 Hz) levels after about 300 scans. In the lower panel – that reports the results for the stochastic DCM – we see same trend of average root square error decreasing with increasing run lengths but it never attains the (heuristic) threshold of 0.1 Hz.

**Fig. 5:**
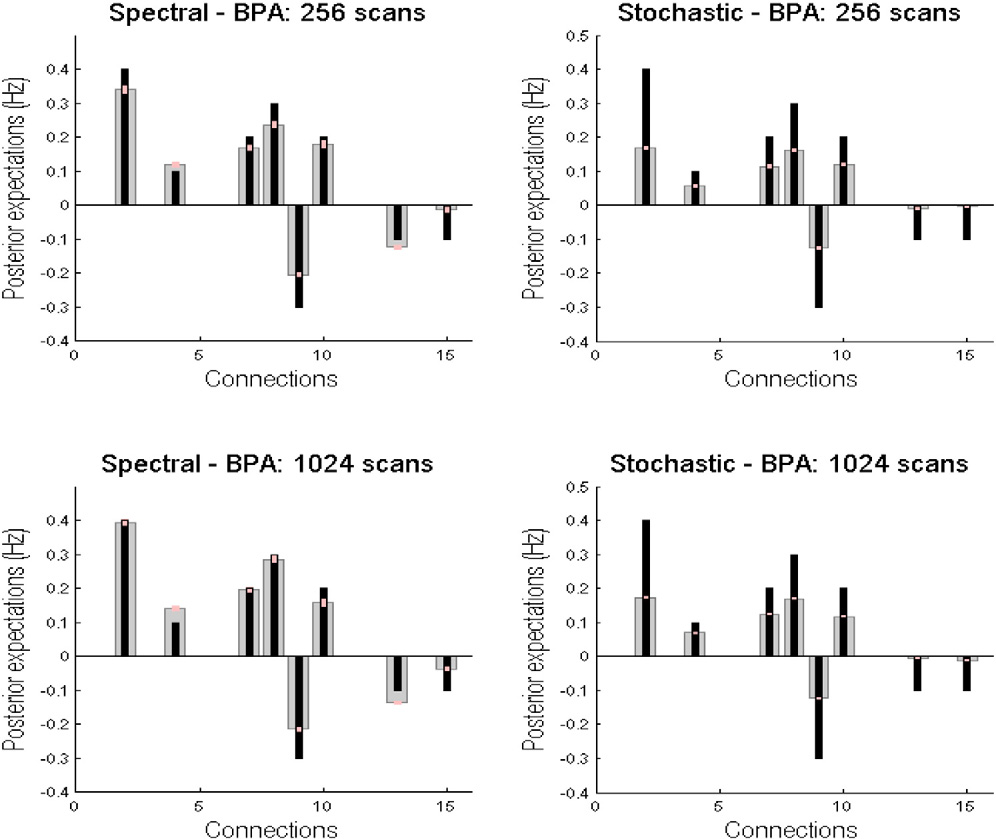
This figure reports the Bayesian parameter averages of the effective connection strengths using the same format as in Fig. 3. Because we have pooled over 32 simulated subjects, the confidence intervals are much smaller (and also note the characteristic shrinkage one obtains with Bayesian estimators). The right column (resp. left column) shows the results for spectral DCM (resp. stochastic DCM) revealing the similarity between the Bayesian parameter averages from long runs (upper panel) and shorter runs (lower panel), of 1024 and 256 scans, respectively.

In Monte-Carlo simulations, we generated the time series of various lengths ranging from 128 time points to 1024 time points with a step size of 128. We simulated 32 realisations (as a proxy for number of participants) for each run length and calculated the root mean squared error. The mean RMS error for each run length (averaging over 32 simulations for each run length) is presented in Fig. 4. For spDCM, RMS error decreases as the number of time points increases. It is satisfying to note that the RMS drops below the typical effective connectivity value of 0.1 Hz, for times series with 384 time points, corresponding to around 13 min of scanning (with a repetition time of 2 s). Whilst the sDCM RMS also decreases with longer time series, the errors for sDCM estimates are greater than those for spDCM, and fail to reach the 0.1 Hz threshold. Whilst the accuracies of the sDCM estimates are impressive, demonstrating the scheme insensitivity to the run length, the estimation accuracy (for this particular graph) was inferior to spDCM. The Bayesian parameter averages (ignoring posterior correlations) of the effective connectivity are shown in Fig. 5 as a function of session length. As has been previously noted (Friston et al., 2014), these Bayesian parametric averages do not change greatly when comparing, for example, run lengths of 256 and 1024. As we are pooling over many simulations, the confidence intervals around these Bayesian parameter averages are fairly small. This suggests that when a sufficient number of participants are used, shorter run lengths may provide reasonably accurate estimates.

In summary, on the basis of the simulations reported here (and many others not shown) it appears that spectral DCM outperforms stochastic DCM in terms of accuracy — as measured by deviation from the true parameters generating data. Having said this, the main difference between the two schemes is a greater shrinkage of Bayesian estimators for the stochastic scheme, which is consistent with the conditional dependencies between hidden states and parameters it has to contend with. In terms of construct validation, it is reassuring that both schemes provide qualitatively similar (internally consistent) estimates — and that both are consistent with the parameters of process generating data.

Although being able to estimate effective connectivity from resting state fMRI may be interesting in and of itself, the usual questions in the setting are about differences in effective connectivity among cohorts, which we now look at more closely.

## Comparative inversions testing for group differences

In this section, we examine whether group differences in effective connectivity can be detected by the two schemes. Group differences were simulated in the following way: Time series of 512 time points were generated for 48 participants using the above 4 node DCM (with 16 connections in total). The first 24 instances used the connectivity matrix given by (13), whilst the second 24 simulations were generated using an altered matrix — the strength of the extrinsic coupling from node 2 to node 3 was made more excitatory (increased from 0.2 to 0.5 Hz). It should be noted that by making this connection stronger, we also break the symmetry in reciprocal connectivity between nodes 2 and 3, hence making it more difficult for model inversion to recover these connections. Additionally, we also introduced a backward connection between nodes 3 and 4 by increasing the inhibition from 0.0 to − 0.3 Hz. We used both Bayesian and classical inference (*t*-tests) to see if these differences can be reliably detected by the two DCMs.

**Fig. 6:**
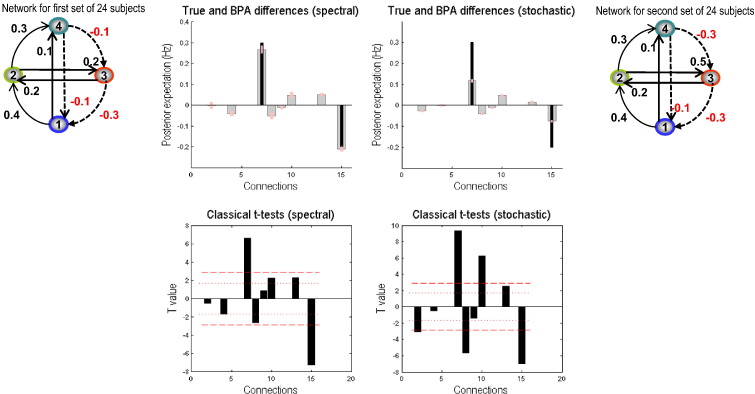
This figure reports the results of a simulated group comparison study of two groups of 24 participants (with 512 scans per participant). The upper row shows the Bayesian parameter averages of the differences using the same format as previous figures. For the spectral DCM (left panel) it can be seen that increases in the extrinsic forward connections from the second to the third region (seventh parameter) has been estimated accurately. Similarly, the decrease in the backward connection from the fourth to the third region is also estimated accurately. For the stochastic DCM (right panel), the estimation of the differences in the two parameter sets is not as accurate — although the direction is detected correctly. The equivalent classical inference — based upon the *t*-statistic is shown on lower row. Here the posterior means from each of 48 subjects were used as summary statistics and entered into a series of univariate *t*-tests to assess differences in group means. The red lines correspond to significance thresholds at a nominal false-positive rate of *p* = 0.05 corrected (solid lines) and uncorrected (broken lines). Clearly the connections with differences survive the corrected threshold for spectral DCM (left panel) whereas for the stochastic DCM (right panel) few other connections are also above threshold.

The Bayesian parameter averages of the group differences for each of the coupling estimates are presented in Fig. 6. The spectral scheme produces both highly accurate and precise estimates of the differences at the two connections manipulated. Any changes in coupling strengths that were not changed were < 0.05 Hz, and can thus be considered a small (and standardised) effect size in quantitative terms. Regarding stochastic DCM, group differences were less accurate but remain qualitatively similar (i.e., they are still in the correct direction). Estimates of differences in other parameters again remained < 0.05 Hz. When applying classical inference (as appears routine in such studies) to the Bayesian parameter averages, both schemes detected significant changes in the two manipulated parameters. The dotted red line corresponds to the thresholds for a nominal level of p = 0.05 uncorrected, whereas the broken red line corresponds to the corrected thresholds for the 16 tests. We used the self-connections for the correction but do not report the (non-significant) differences. For spectral DCM, both forward and backward connections are highly significant, even after correcting for multiple comparisons, and no other connection reached uncorrected threshold. Regarding stochastic DCM, although both backward and forward connections are still detected as the two most statistically significant connections, small changes estimated in a few other connections were found to be significant at corrected statistical thresholds, suggesting that sDCM is sensitive to group differences, but perhaps not as specific as spDCM.

**Fig. 7:**
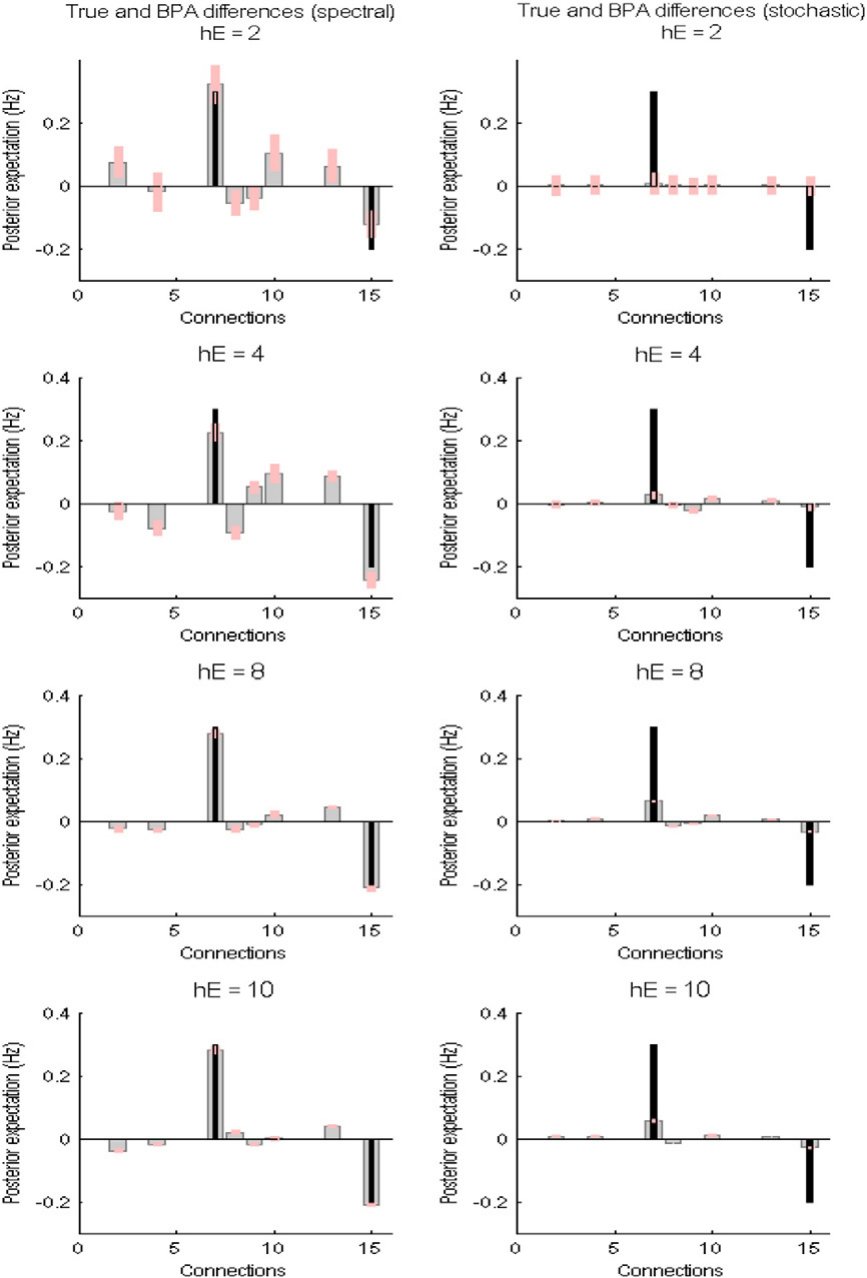
This figure reports the results of changing the priors on measurement noise when characterising group differences for both spectral and stochastic DCMs. The left column shows the Bayesian parameter averages of the differences for spectral DCM and the right column for stochastic DCM — using the same format as in the previous figures. For these results, we kept the prior covariance of the (log) precision parameters constant whilst varying the prior expectation of (log) precision parameters within the range of 2 and 10 with a step size of 2 (except the value of 6 for which the results are already reported in Fig. 6).

We further investigated what effect changing the priors on measurement noise has when characterising group differences. The more precise belief that measurement noise is small may cause stochastic DCM to increase estimated neuronal fluctuations, which may shift parameter estimates towards their true values — and make the performance of the stochastic DCM approach that of spectral DCM. The results in Fig. 6 were obtained with a default value of 6 for the prior expectation of (log) noise precision and 1/128 for its prior covariance. To evaluate the effect of measurement noise on the group differences we kept the (hyper) prior covariance constant at 1/128 whilst varying the (hyper) prior expectation from 2 to 10 in steps of 2. The results are reported in Fig. 7. We notice that increasing the expected noise (log) precision provided increasingly accurate estimates for spectral DCM, whereas the most accurate estimates for stochastic DCM were recovered whilst using the default value of 6.

**Fig. 8:**
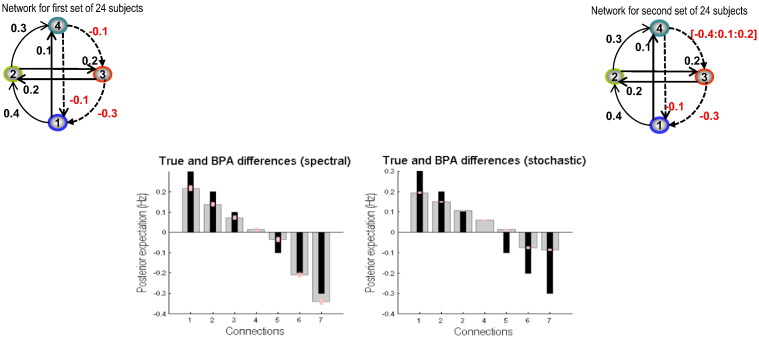
This figure reports the sensitivity of both schemes based on simulations of the sort reported in the previous figure. For these results, we varied the connection from node 4 to node 3 in the range of − 0.4 to 0.2 with a step size of 0.1 such that group differences were in the range of 0.3 to − 0.3. The left panel shows the Bayesian parameter averages of the differences for spectral DCM using the same format as previous figures — and the right panel shows the results for stochastic DCM.

Finally, we evaluated the sensitivity of the two methods in detecting group differences when we varied one connection over a range of values. The results are reported in Fig. 8. In this simulation, we kept all the connectivity parameters fixed across both groups of 24 subjects, except the connection from node 4 to node 3. We varied this connection in second set of 24 subjects between − 0.4 and 0.2 in steps of 0.1. This changes the connection from being highly inhibitory to excitatory. The consequent group differences are in the range of 0.3 to − 0.3 with a sign change to make it more interesting. We see that spectral DCM performed better than stochastic DCM which usually underestimated the differences. Importantly, spectral DCM was very efficient in recovering the sign change, whereas stochastic DCM failed when the change was subtle. As the change becomes larger stochastic DCM improved although not attaining the sensitivity of spectral DCM.

## An empirical application

There has been interest in the connectivity within the *default mode network* (DMN) — a distinct brain system that is activated when an individual engages in introspection like mindwandering or daydreaming. The DMN comprises part of the medial prefrontal cortex (mPFC), posterior cingulate cortex (PCC) and parts of inferior parietal lobe and superior frontal regions. The regions within the DMN are highly interconnected at rest and show developmental changes during adolescence (Fair et al., 2008), whilst the coherence among DMN regions diminishes in healthy ageing (Andrews-Hanna et al., 2007). Furthermore, alterations in the functional connectivity among DMN regions are seen in Alzheimer's disease (Greicius et al., 2004), and schizophrenia (Calhoun et al., 2008; Harrison et al., 2007). However, there are relatively very few studies characterising either effective or directed functional connectivity within the DMN. Examples of such studies include Granger causality modelling in healthy controls (Jiao et al., 2011; Zhou et al., 2011), Bayesian network analysis in AD (Li et al., 2013; Wu et al., 2011), and DCM in healthy controls (Di and Biswal, 2014; Li et al., 2012).

We compared the model structure and posterior coupling estimates among a subset of nodes comprising the DMN using stochastic DCM and spectral DCM. Data were downloaded from the open access dataset from the FC1000 project. This dataset contains 22 adults (12 males) with a mean age of 29 years. Scanning was performed at the University of Oxford Centre for Clinical Magnetic Resonance Research using a 3-T Siemens Trio scanner with a 12-channel head coil. Whole-brain functional imaging was performed using a gradient echo EPI sequence (TR = 2000 ms, TE = 28 ms, flip angle = 89°, field of view = 224 mm, voxel dimension = 3 × 3 × 3.5 mm, acquisition time = 6 min 4 s). High-resolution 3D T1-weighted MRI scans were acquired using a magnetization-prepared rapid gradient echo sequence (TR = 2040 ms, TE = 4.7 ms, flip angle = 8°, field of view = 192 mm, voxel dimension = 1 mm isotropic, acquisition time = 12 min). Participants were instructed to lie in dimmed light with their eyes open, thinking of “nothing in particular” and not to fall asleep. From the functional data containing 180 consecutive image volumes per participant, the first five volumes from each participant were removed. Data were pre-processed in the normal way: data were realigned, normalized to MNI space, and spatially smoothed using a 6 mm (FWHM) Gaussian kernel. A GLM containing only movement (confound) regressors was constructed and inverted. An adjusted time series from the lateral ventricle was included in subsequent GLMs as an additional confound.

**Fig. 9:**
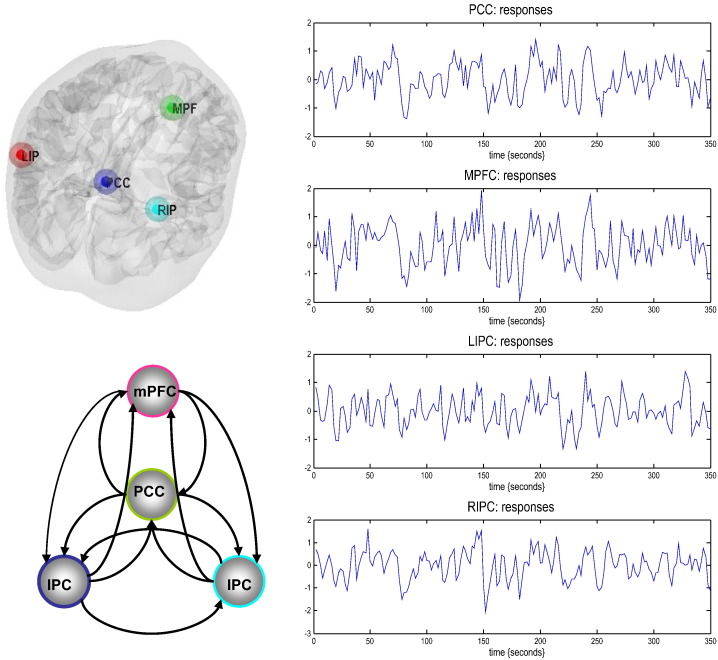
Summary of empirical time series used for the illustrative analysis. The time series (right-hand panels) from four regions are the principal eigenvariates of regions identified using seed connectivity analyses (upper left insert) for a typical subject. These time series we used to invert the DCMs (both spectral and stochastic) with the (fully-connected) architecture shown in the lower left panel.

To identify nodes of the DMN, the resting state was modelled using a GLM containing a discrete cosine basis set with frequencies ranging from 0.0078 to 0.1 Hz (Fransson, 2005; Kahan et al., 2014), in addition to the aforementioned nuisance regressors. Data were high-pass filtered to remove any slow frequency drifts (< 0.0078 Hz) in the normal manner. An F-contrast was specified across the discrete cosine transforms (DCT), producing an SPM that identified regions exhibiting BOLD fluctuations within the frequency band. Our DMN graph comprised of four nodes; the posterior cingulate cortex (PCC), the right and left intraparietal cortex (R/LIPC), and the medial prefrontal cortex (mPFC). The PCC node was identified using this GLM: the principal eigenvariate of a (8 mm radius) sphere was computed (adjusted for confounds), centred on the peak voxel of the aforementioned F-contrast. The ensuing region of interest was masked by a (8 mm radius) sphere centred on previously reported MNI coordinates for the PCC [0, − 52, 26] (Di and Biswal, 2014). The remaining DMN nodes were identified using a standard seed-based functional connectivity analysis, using the PCC as the reference time series in an independent GLM containing the same confounds. A *t*-contrast on the PCC time series was specified, and the resulting SPM was masked by spheres centred on previously reported coordinates for the RIPC [48, − 69, 35], LIPC [− 50, − 63, 32], and mPFC [3, 54, − 2] (Di and Biswal, 2014). The principal eigenvariate from a (8 mm radius) sphere centred on the peak *t*-value from each region was computed for each region and corrected for confounds. The time series extracted from each of the four regions – for typical subject – are shown in Fig. 9.

For each participant, a fully connected DCM, with no exogenous inputs, was specified. The DCM was inverted using both stochastic DCM, and spectral DCM. Following inversion of the full model, post-hoc model optimization was employed to search the model space for reduced models with the highest model evidence (Friston et al., 2011).

**Fig. 10:**
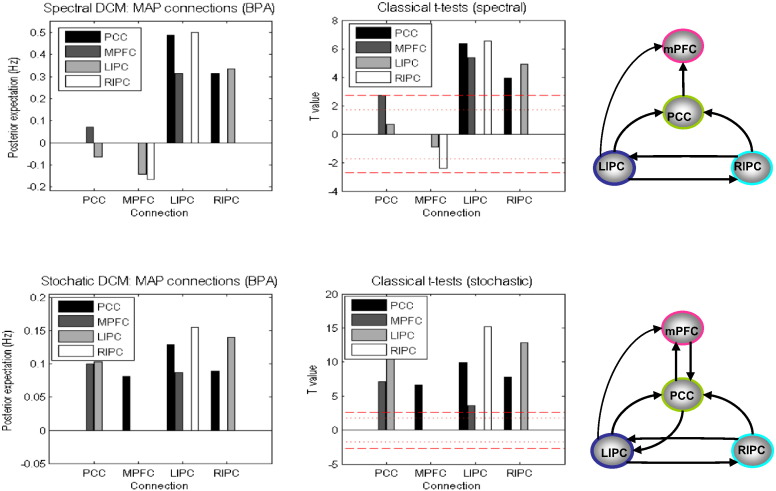
This figure summarises the results of model inversion using the model and data of previous figure. We only show the results for connections with non-trivial connection strengths greater than 0.5 Hz (and omit self-connections for simplicity). The upper row shows the results for the spectral DCM in same format in previous figures for simulated data. The leftmost panel shows the Bayesian parametric averages over 22 subjects. The middle panel shows the results of classical *t*-tests reporting *t*-statistics for each connection, whereas the right panel shows only those edges on the graph that survive the corrected threshold in the middle panel. The lower row reports the results for stochastic DCM in the same format as for the spectral DCM.

Post-hoc model optimization found the fully connected model to have the largest free energy, consistent with previous similar analyses (Li et al., 2012). Bayesian parameter averaging (BPA) was then used to calculate the expected posterior connectivity estimates, and posterior confidence intervals. The concordance between stochastic and spectral results was subsequently examined qualitatively. In Fig. 10, we show the results of both the stochastic and spectral DCM schemes. The left columns show the results of BPA. To focus on non-trivial connections, we only report connection strengths that exceed 0.05 Hz in strength. We also omit self-connections in these plots for simplicity. BPA results are fairly consistent when compared between the two schemes, especially the connections originating from the bilateral intraparietal cortex. There is some disagreement in some other connections originating from the PCC and mPFC. Furthermore, coupling strengths from stochastic DCM are relatively smaller in magnitude than those from the spectral DCM, which is in accordance with previous simulations demonstrating that stochastic DCM tends to underestimate the effective connectivity (see also above). In the middle column, we show the results of classical *t*-tests to see which of the connections are significantly different from zero. In the last column, only the significant connections surviving the corrected threshold (the broken red line) are shown.

Previous studies of the coupling between regions within DMN have produced some inconsistent results. For example (Di and Biswal, 2014) (using DCM), and (Jiao et al., 2011; Zhou et al., 2011) (using Granger causality) found a causal influence from mPFC to PCC but not vice versa. We see this connection to be present in stochastic DCM but not in spectral DCM. Li et al. (2012) (using stochastic DCM) showed an influence from PCC to mPFC which we also see in both schemes. A more consistent finding is that bilateral IPC drives PCC (Di and Biswal, 2014; Jiao et al., 2011; Zhou et al., 2011), which is also the case in both of our DCM results. We also see that mPFC is driven by LIPC, again consistent with most previous studies (Di and Biswal, 2014; Jiao et al., 2011; Zhou et al., 2011). Di and Biswal (2014) and Jiao et al. (2011) reported that an influence from RIPC to mPFC that we failed to detect in either of the DCMs, is in line with previous studies (Zhou et al., 2011). Interestingly, there is a reciprocal connection between bilateral IPC in both DCM models, also reported in Li et al. (2012). Zhou et al. (2011) found influence from RIPC to LIPC but not vice versa, and Di and Biswal (2014) and Jiao et al. (2011) did not find any interaction at all between bilateral IPC.

The empirical demonstrations in this section should not be over interpreted. Their purpose is to illustrate the application of the procedures described to real data. For example, the fact that the nodes included in the DCM were identified using correlations with a seed region may preclude the identification of subgraphs that show a sparse connectivity. In principle, similar procedures can be applied with relatively unbiased region identification; using, for example, independent component analysis (as suggested by one of our reviewers). Bayesian model comparison may then reveal sparser connectivity.

**Fig. 11:**
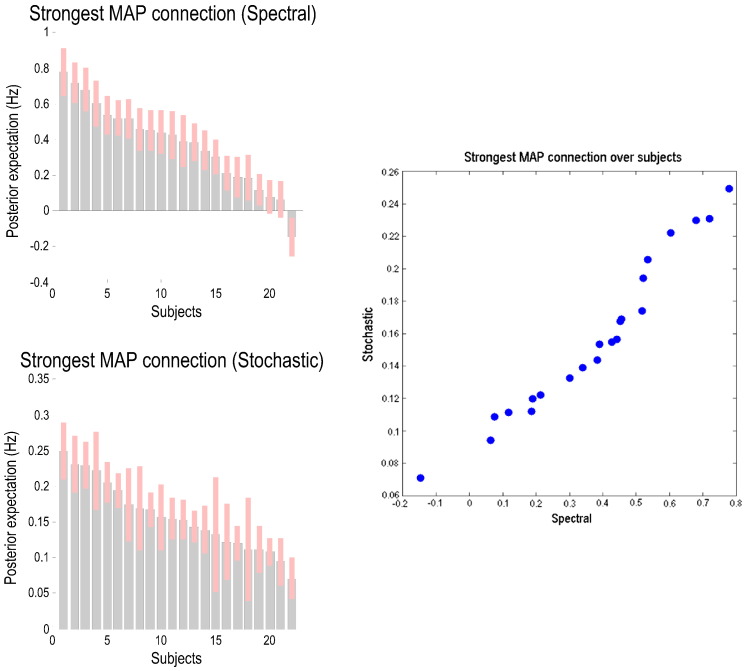
This figure plots the distribution of the posterior expectations of the two schemes over subjects for the strongest connection from left IPC to right IPC (see Fig. 10). The posterior expectations were ranked in descending order. The upper left panel shows the posterior expectations (light grey bars) for the spectral DCM with superimposed confidence interval (pink bars). A similar plot for stochastic DCM is shown in the lower left panel. We also show scatter plot of the posterior expectations over subjects for the two schemes.

Finally, to assess the comparative reproducibility of spectral and stochastic DCM estimates we examined the distribution of connection strengths (and their respective confidence intervals) for the most significant connection (from left to right IPC) over subjects — ranked from the highest to the lowest posterior expectation. The two distributions for spectral (upper left) and stochastic estimators (lower left) are shown in Fig. 11. It can be seen that there is a remarkable consistency over subjects for both estimators — except that one participant's connection was in the opposite direction for spectral DCM. Furthermore, again we see that stochastic estimators are shrunk towards the prior expectations (of zero) relative to the spectral estimators. We also included a scatter plot of the two distributions (right panel) over participants which showed high correlations over participants' estimates.

## Discussion

In this technical note we address the construct validation of deterministic spectral DCM for the resting state fMRI by comparing it to a stochastic DCM scheme for estimating effective connectivity from resting state fMRI datasets. Spectral DCM is particularly useful for interrogating group differences in effective connectivity, or the time constants of the neural fluctuations, where the latter can be assessed in the form of the autocorrelation function of endogenous neuronal fluctuations.

The disadvantage of this deterministic DCM for cross spectra rests on linear system analysis, which precludes state or time-dependent changes in effective connectivity (Breakspear, 2004). In other words, unlike deterministic DCM for time series, one cannot model – in a simple way – changes in effective connectivity caused by experimental manipulations or nonlinear state-dependencies. Having said this, most applications of resting state fMRI focus on group differences—as opposed to state or set-dependent differences that are usually modelled with time-dependent (e.g., bilinear) changes in coupling. Furthermore, spectral DCM is computationally very efficient, compared to stochastic DCM. This is because spectral DCM does not require the estimation of the hidden states per se. Compared to stochastic DCM, the inversion of spectral DCM takes about 10 s per iteration for a 10 node graph, with convergence achieved usually between 16 and 64 iterations. It is noteworthy that the inversion of spectral DCM is even faster than conventional deterministic DCM; since it does not require the integration of differential equations. The computational efficiency could further be increased by using more efficient (e.g., adjoint) gradient computation methods. DCM uses finite differences to compute the gradients during the optimization of free energy to update model parameters. Using advanced gradient computation methods based on the adjoint could further improve the computational efficiency of DCM (Sengupta et al., 2014).

Furthermore, using prior constraints that bound the number of free parameters, the Bayesian inversion of large graphs (nodes > 32) can be made computationally very efficient. This simple but graceful solution uses prior (functional connectivity) modes to reduce the dimensionality of the problem in an informed way (Seghier and Friston, 2013). In future, we foresee employing such constraints with spectral DCM to invert large DCMs of resting state fMRI data. Finally, as we have noted previously — the ability to estimate weighted and directed adjacency matrices summarising functional brain architectures also opens the door to graph theoretic analyses that may leverage important advances in network theory (Rubinov and Sporns, 2010).

## Footnotes

1 Strictly speaking, the hidden states include both neuronal and haemodynamic states; however, for simplicity, we will ignore haemodynamic states in this paper.
